# Supplementary material for: Two mouse models reveal an actionable PARP1 dependence in aggressive chronic lymphocytic leukemia
Source: Nat Commun. 2017 Jul 28;8:153. doi: 10.1038/s41467-017-00210-6 (PMC5532225; doi:10.1038/s41467-017-00210-6)
Supplement: Supplementary file 1 — Supplementary Information [file 41467_2017_210_MOESM1_ESM.pdf]

Title of file for HTML: Supplementary Information
Description: Supplementary Figures and Supplementary Tables

Title of file for HTML: Peer Review File
Description:

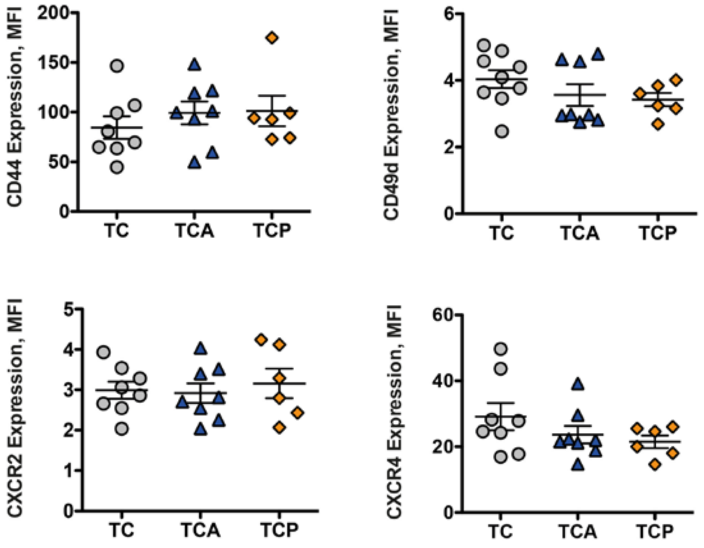

Supplementary Fig. 1: Expression levels of homing factors on the surface of CD5$^+$/CD19$^+$ splenocytes from TC, TCA and TCP animals. Illustrated are the median fluorescence intensities (MFI) of CD44, CD49D, CXCR2 and CXCR4 from CD5$^+$/CD19$^+$ leukemic cells isolated from spleens of TC, TCA and TCP mice. No significant differences were detected (Welch's t-test). Error bars represent SEM.

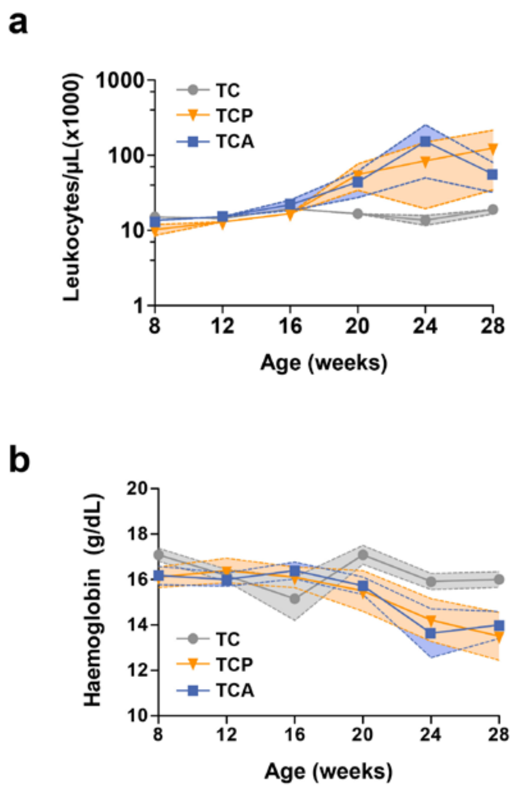

Supplementary Fig. 2: Development of leukocytosis and anemia in TC, TCA and TCP animals. Leukocyte counts **(a)** and haemoglobin concentrations **(b)** in the peripheral blood of TC, TCA and TCP mice are plotted over time (n = 10 animals per group). Envelopes respresent SEM.

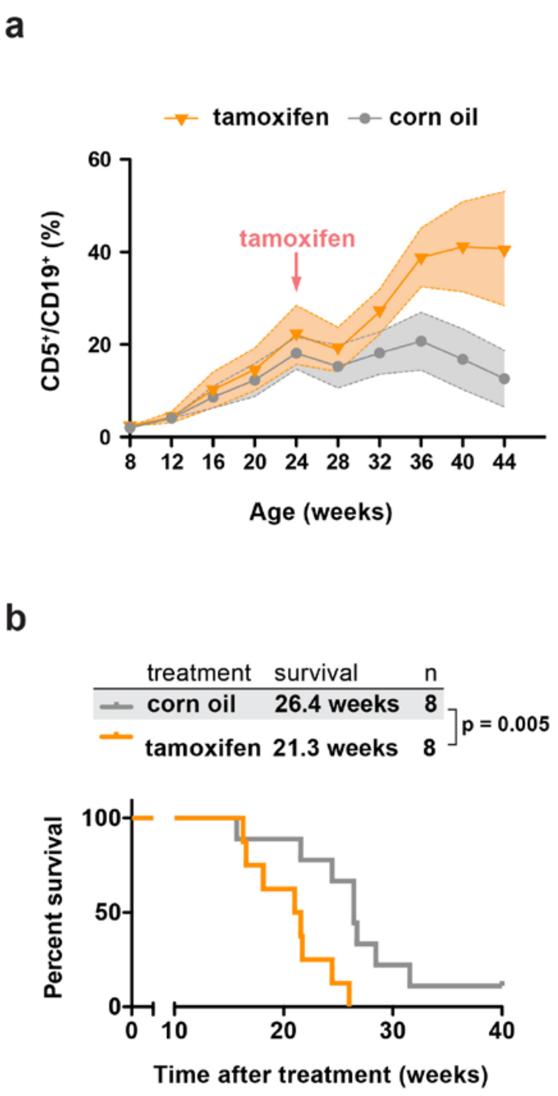

Supplementary Fig. 3: The inducible loss of *Trp53* in leukemic TC mice leads to an accelerated disease progression and reduced overall survival. Leukemic *Eμ:TCL1;Cd19*^{CreERT2}*;Trp53*$^{fl/fl}$ mice were injected intraperitoneally with either 4OH-tamoxifen or corn oil for five consecutive days at 24 weeks of age. The induction of bi-allelic *Trp53* deletion results in an accelerated accumulation of leukemic $CD5^+$/$CD19^+$ cells in the peripheral blood **(a)** and a reduced overall survival, compared to corn oil-treated control animals **(b)**. Envelopes respresent SEM. n = 8 per cohort.

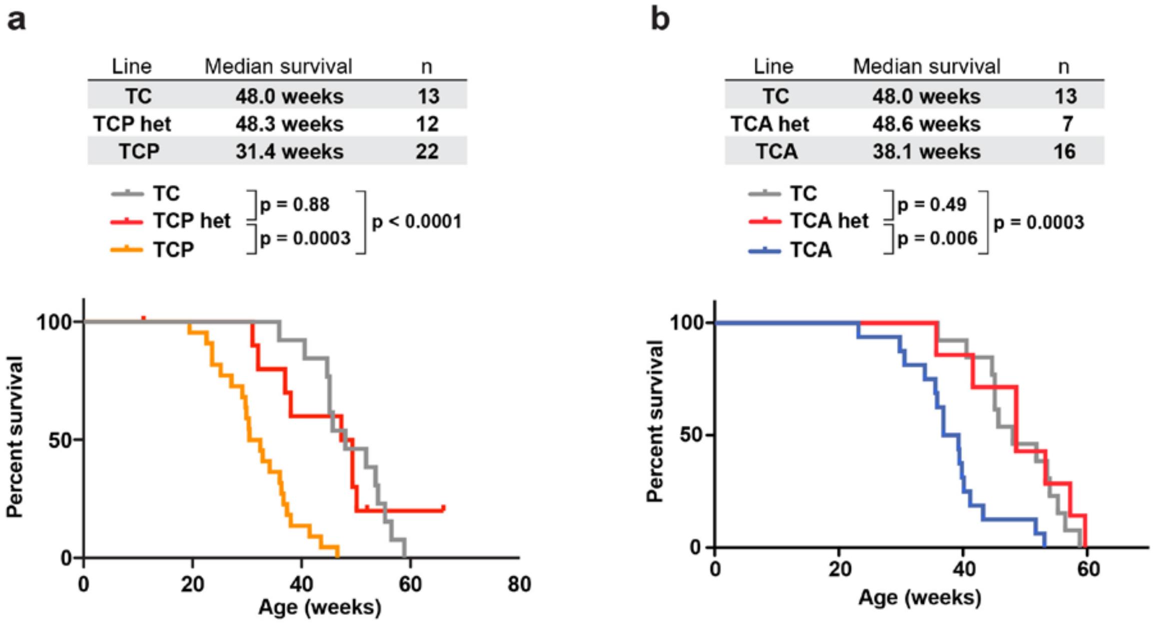

Supplementary Fig. 4: Heterozygous loss of *Trp53* or *Atm* does not cause accelerated disease progression or reduced overall survival. The overall survival of *Eμ:TCL1;Cd19*$^{Cre/wt}$*;Trp53*$^{fl/wt}$ **(a)** and *Eμ:TCL1;Cd19*$^{Cre/wt}$*;Atm*$^{fl/wt}$ **(b)** mice is not significantly different from the survival of *Eμ:TCL1;Cd19*$^{Cre/wt}$ control animals. **a)**, **b)** log-rank test.

a

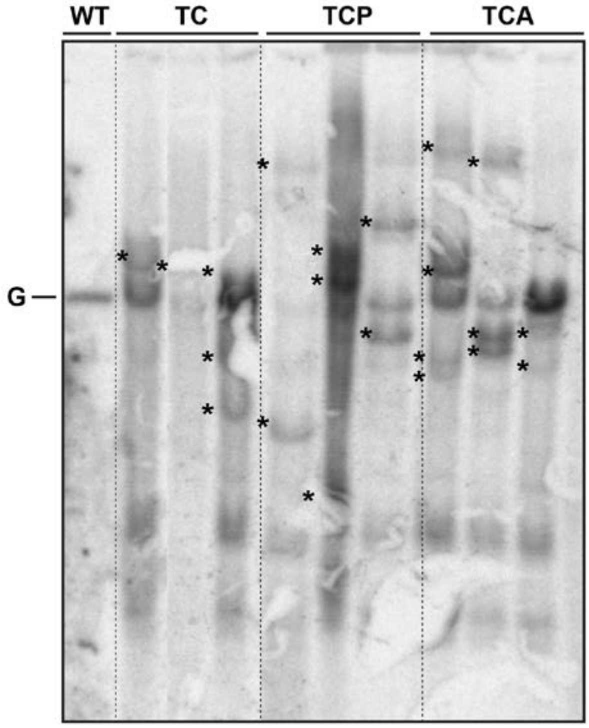

b

Sample	*Ighv*	*Ighd*	*Ighj*	*ident. %*	*in-frame*	*stop codon*	*pot. func.*
1	2-9-1	2-3	2	100	yes	no	yes
2	3-1	1-1	4	99.57 (234/235)	yes	no	yes
3	1-55	1-1	2	100	yes	no	yes
4	2-2	???	4	100	no	no	no
5	11-2	2-5	1	100	yes	no	yes
6	5-15	2-5	2	100	yes	no	yes

Supplementary Fig. 5: TC, TCA and TCP mice develop oligoclonal, *Igh*-unmutated disease. a) Southern blot analysis of EcoRI-digested DNA from CLL cells isolated from infiltrated spleens (n = 3 for each genotype). Asterisks mark clonal rearrangements. ‘G’, germline sequence. **b)** Direct sequencing of clonal rearrangements (samples 1, 2: TC; samples 3, 4: TCA; samples 5, 6: TCP) reveals an unmutated *Igh* status in all three genotypes.

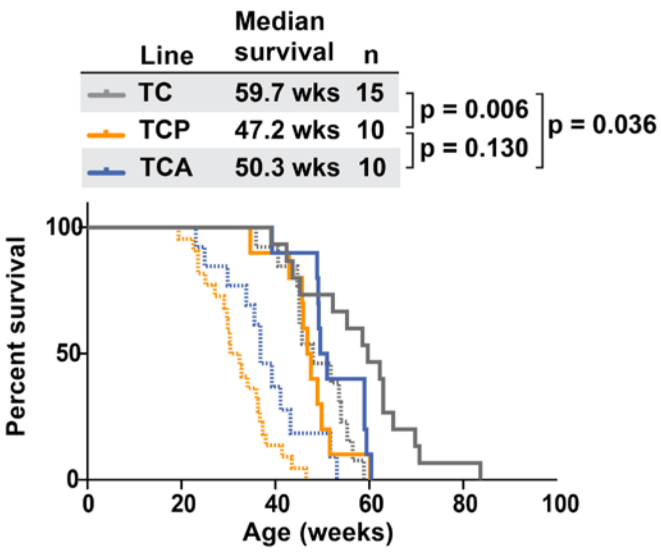

Supplementary Fig. 6: Cyclophosphamide treatment leads to a significant survival benefit in TC, TCA and TCP animals. Animals of all three genotypes (TC [n = 15], TCP [n = 10] and TCA [n = 10]) were treated with cyclophosphamide (200 mg/kg, i.p., 4 injections, once weekly). Cyclophosphamide-treated cohorts are depicted as solid lines, untreated controls as dashed lines. Indicated are the p values (log-rank test) for the comparison of the treated groups. The survival benefit compared to the respective untreated control is significant for all three lines (TC: $p = 0.004$, TCP: $p < 0.0001$, TCA: $p = 0.004$, log-rank test).

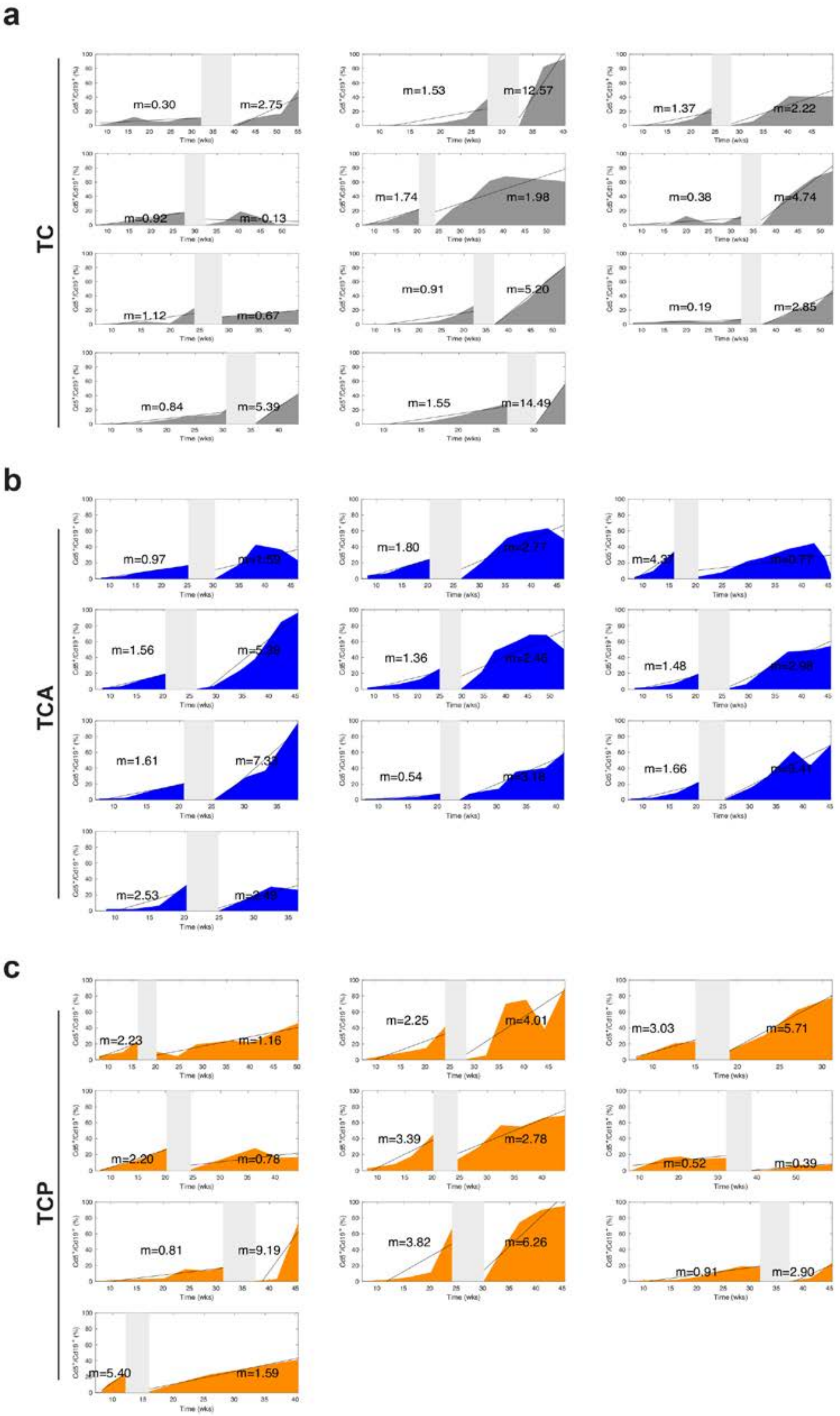

Supplementary Fig. 7: Longitudinal monitoring of leukemic burden before and after cyclophosphamide treatment in TC, TCA and TCP animals. The percentage of $CD5^+$/$CD19^+$ cells within the $CD45^+$ lymphocyte gate in the peripheral blood of individual TC (n = 11), TCA (n = 10) and TCP animals (n = 10) is plotted over time.

Grey areas mark the time period of therapeutic intervention (200mg/kg cyclophosphamide, i.p., once weekly, four injections). The data before and after treatment were linearly fitted (black lines). The slope of the fit (representing the change in leukemic burden in percent points per week) is annotated in the graphs and summarized in **Fig. 3 h, i**.

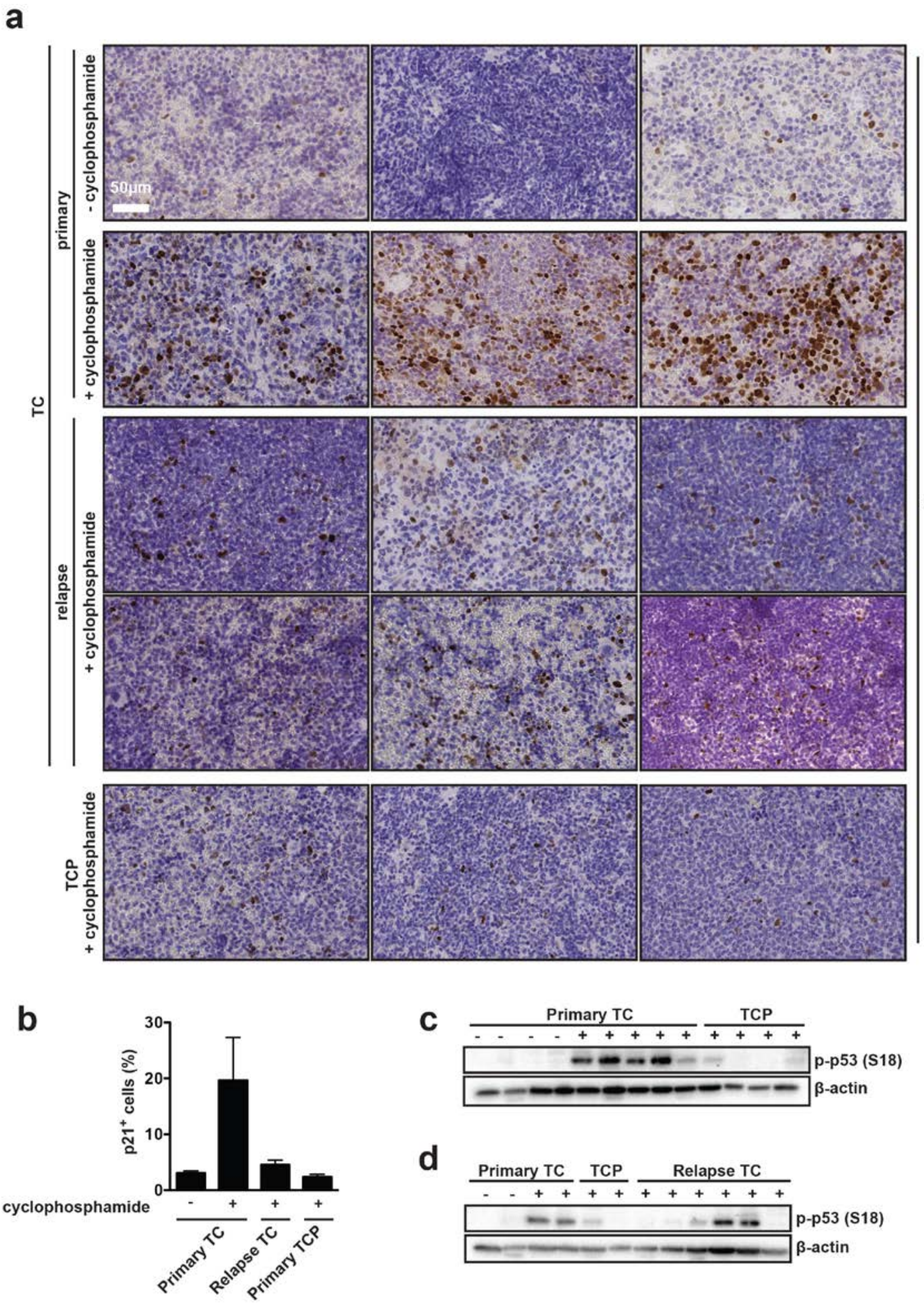

Supplementary Fig. 8: Induction of p21 following cyclophosphamide treatment *in vivo*. Leukemic chemotherapy-naïve animals and mice that had relapsed following four cycles of cylcophoshamide were injected with a single dose of cyclophosphamide (200 mg/kg, i.p.) and sacrificed 12 hours later. Immunohistochemical stainings for p21 within the leukemia-infiltrated spleens of

these animals are shown in **a)**. Each tile represents an individual animal. Untreated TC and cyclophosphamide-treated TCP spleens were used as controls. **b)** Quantification of the IHC stainings shown in **a)**. **c)** and **d)** Lysates were generated from splenocytes of animals with primary and relapsed lesions. Animals were either left untreated ('-') or injected with cyclophosphamide 12 hours prior to sacrifice ('+'). Error bars represent SEM. Scale bar: 50 µm.

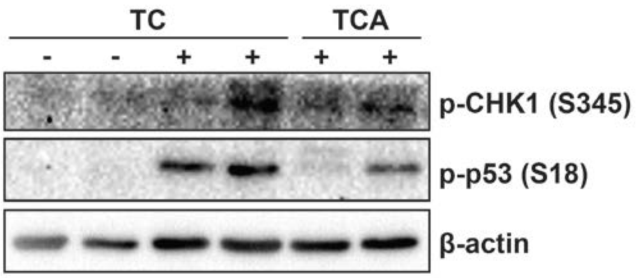

Supplementary Fig. 9: Activation of the ATR/CHK1/p53 pathway after acute cyclophosphamide challenge in TCA animals. Leukemic TC and TCA animals with enlarged spleens were injected with cyclophosphamide (150 mg/kg, i.p.) and sacrificed 12 hours after. Lysates generated from isolated splenocytes were analyzed for phosphorylated CHK1 (Ser345) and phosphorylated p53 (Ser18) by western blot.

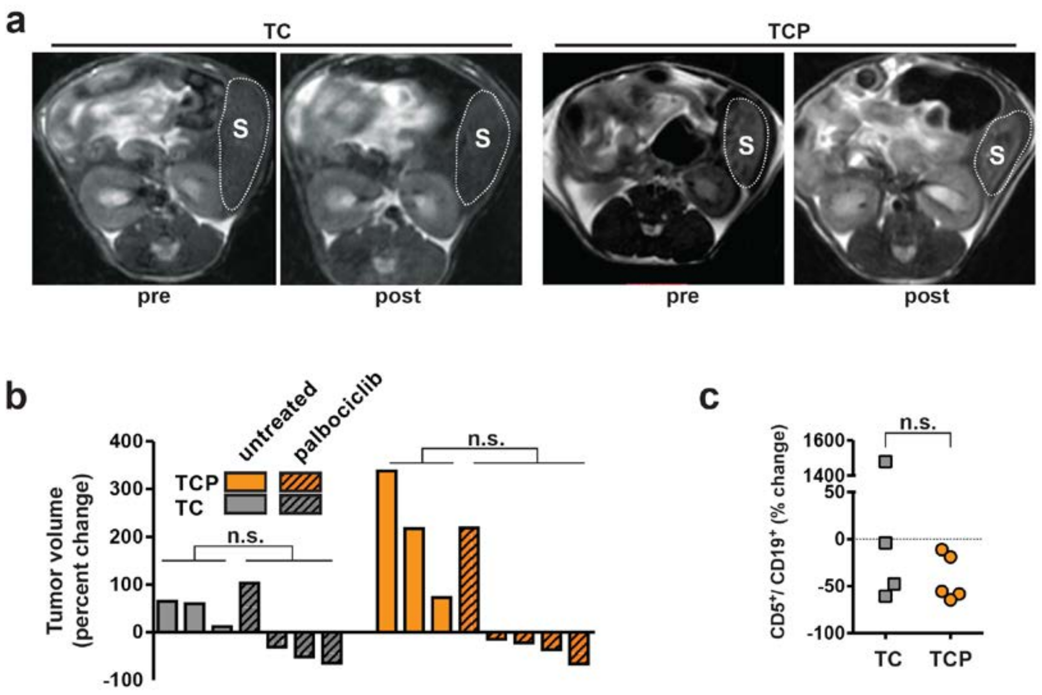

Supplementary Fig. 10: Palbociclib treatment causes a mild reduction of leukemic burden in the spleens and peripheral blood of TC and TCP mice. TC and TCP mice (TC: n = 4, TCP: n = 5) were treated with the CDK4/6 inhibitor palbociclib (a daily dose of 150 mg/kg by oral gavage for 14 consecutive days). Spleen volumes before and after treatment were assessed by volumetrisation of MRI scans and are illustrated in **a)** and **b)**. The percentual change of the leukemic burden in the peripheral blood was assessed by flow cytometric identification of the CD5+/CD19+ population in the peripheral blood before and after the two weeks of treatment **(c)**. **b)** and **c)** Welch's t-test.

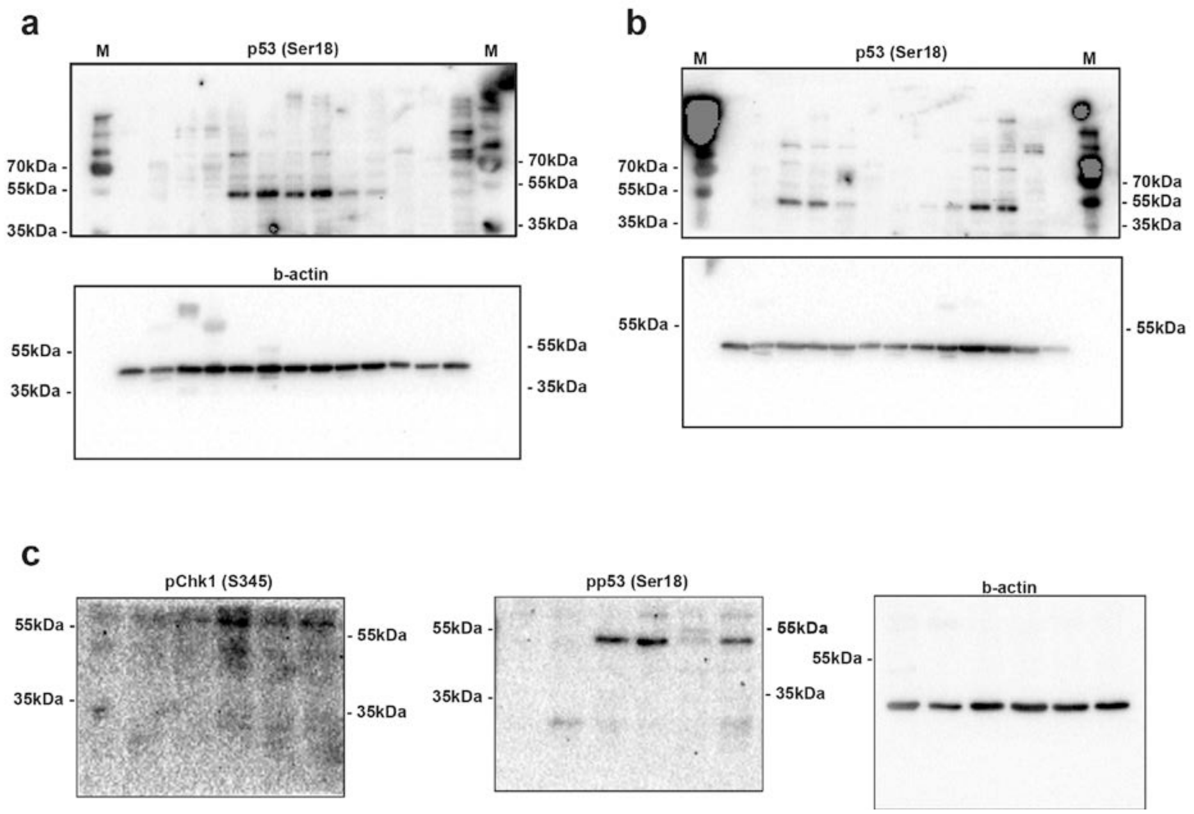

Supplementary Fig. 11: Uncropped immunoblotting images. Shown are uncropped images of the immunoblotting membranes used in **(a) Supplementary Fig. 8c**, **(b) Supplementary Fig. 8d** and **(c) Supplementary Fig. 9**.

sample type	*min coverage*	*mean coverage*
primary	23708	47275
primary	28205	61000
primary	28351	76299
primary	29655	73432
primary	29681	66359
primary	30024	64005
primary	36138	76888
primary	38393	83424
relapse	23220	53466
relapse	23933	63483
relapse	23967	66124
relapse	27951	73384
relapse	28072	91649
relapse	29053	72033
relapse	29135	69677
relapse	29219	78679
relapse	35171	78708
relapse	35269	74813
relapse	38072	95255

Supplementary Table 1: Trp53 sequencing coverage. Minimum and mean sequencing coverage for each sample sequenced for *Trp53*.

gene symbol	*p value*	*log2(fold change)*
**TC untreated vs. treated**		
Apaf1	7.22E-03	0.75
Bax	9.25E-04	1.60
Bbc3	9.21E-03	0.48
Bub1	3.12E-02	-1.52
Bub1b	3.13E-02	-0.91
Ccna2	4.35E-02	-1.31
Ccnb1	1.51E-02	-1.49
Ccnb2	2.72E-02	-1.36
Ccng1	4.03E-03	0.91
Cdkn1a	5.51E-03	2.79
E2f3	3.34E-02	-0.50
Ei24	5.29E-03	1.29
Gtse1	2.17E-02	0.60
Mcm5	3.67E-02	-0.78
Mdm2	7.89E-03	1.06
Mdm4	1.15E-03	0.46
Plk1	2.00E-02	-1.23
Rbl2	1.06E-02	1.00
Zmat3	2.03E-02	1.00
**TCA untreated vs. treated**		
Anapc10	1.74E-02	-0.37
Bax	2.29E-03	0.87
Ccng1	1.20E-03	0.73
Cdkn1a	1.80E-02	2.36
Cul1	2.69E-02	-0.33
Cycs	2.65E-04	-0.34
Ddb2	2.01E-02	0.43
E2f3	3.30E-02	-0.56
Ei24	2.61E-05	0.74
Mdm2	1.02E-02	0.56
Prkdc	5.34E-03	-0.36
Rbl2	5.19E-03	0.43
Rbx1	6.47E-03	-0.45
Rprm	1.30E-03	0.39
Rrm2b	4.52E-02	-0.44
Ywhag	3.23E-02	-0.40
**TCP untreated vs. treated**		
Ccne2	3.27E-02	0.98
Cdc6	2.05E-02	0.69
Ep300	1.37E-03	-0.31
Orc6	1.82E-02	0.46

Supplementary Table 2: Differentially expressed genes of the KEGG pathways “p53 signaling pathway” and “cell cycle”.

gene symbol	*p value*	*log2(fold change)*
**TC vs. TCA**		
Parp1	4.2E-02	0.53
Pold3	2.2E-02	0.39
**TC vs. TCP**		
Smug1	4.6E-02	0.41

Supplementary Table 3: Differentially expressed genes of the KEGG pathway “base excision repair”.

forward primer	*reverse primer*
GGCCCCTGTCATCTTTTGT	GCATTGAAAGGTCACACGAA
CGTTCTCTCTCCTCTCTTCCAG	TGCTGTGACTTCTTGTAGATGG
GTGCCCTGTGCAGTTGTG	CCATCACCATCGGAGCAG
GCCATGGCCATCTACAAGAA	TAAGTCAGAAGCCGGGAGAT
TCCCGGCTTCTGACTTATTC	AGACCTCGGGTGGCTCAT
GCTCCTCCCCAGCATCTTAT	GACGCACAAACCAAAACAAA
ACCTGGATCCTGTGTCTTCC	CGCCTTCCTACCTGGAGTCT
AACCGCCGACCTATCCTTAC	TGGAACAGAAACAGGCAGAA
TGCTGGTCCTTTTCTTGTCC	AGTTCAGGGCAAAGGACTTC
GAGAGACCGCCGTACAGAAG	GTGACTTTGGGGTGAAGCTC

Supplementary Table 4: ***Trp53* sequencing primers**.
